# Bayesian and frequentist approaches to sequential monitoring for futility in oncology basket trials: A comparison of Simon’s two-stage design and Bayesian predictive probability monitoring with information sharing across baskets

**DOI:** 10.1371/journal.pone.0272367

**Published:** 2022-08-02

**Authors:** Alexander Kaizer, Emily Zabor, Lei Nie, Brian Hobbs

**Affiliations:** 1 Department of Biostatistics and Informatics, Colorado School of Public Health, University of Colorado-Anschutz Medical Campus, Aurora, CO, United States of America; 2 Department of Quantitative Health Sciences & Taussig Cancer Institute, Cleveland Clinic, Cleveland, OH, United States of America; 3 Division of Biometrics II, Office of Biostatistics, Center for Drug Evaluation and Research, U.S. Food and Drug Administration, Silver Spring, MD, United States of America; 4 Department of Population Health, University of Texas-Austin, Austin, TX, United States of America; UNITED STATES

## Abstract

This article discusses and compares statistical designs of basket trial, from both frequentist and Bayesian perspectives. Baskets trials are used in oncology to study interventions that are developed to target a specific feature (often genetic alteration or immune phenotype) that is observed across multiple tissue types and/or tumor histologies. Patient heterogeneity has become pivotal to the development of non-cytotoxic treatment strategies. Treatment targets are often rare and exist among several histologies, making prospective clinical inquiry challenging for individual tumor types. More generally, basket trials are a type of master protocol often used for label expansion. Master protocol is used to refer to designs that accommodates multiple targets, multiple treatments, or both within one overarching protocol. For the purpose of making sequential decisions about treatment futility, Simon’s two-stage design is often embedded within master protocols. In basket trials, this frequentist design is often applied to independent evaluations of tumor histologies and/or indications. In the tumor agnostic setting, rarer indications may fail to reach the sample size needed for even the first evaluation for futility. With recent innovations in Bayesian methods, it is possible to evaluate for futility with smaller sample sizes, even for rarer indications. Novel Bayesian methodology for a sequential basket trial design based on predictive probability is introduced. The Bayesian predictive probability designs allow interim analyses with any desired frequency, including continual assessments after each patient observed. The sequential design is compared with and without Bayesian methods for sharing information among a collection of discrete, and potentially non-exchangeable tumor types. Bayesian designs are compared with Simon’s two-stage minimax design.

## 1. Introduction

Progress in cancer biology and immunology continue to clarify our understanding of cancer mechanisms. This has produced actionable therapeutic targets that extend treatment options for patients beyond conventional cytotoxic therapies. These advances have given rise to the development of biomarker-targeted treatments that transcend traditional classification criteria based on tissue histology. Sosman et al., for example, identified that multiple tumor types harboring *BRAF*^*V*600*E*^ mutations respond to Vemurafenib [1–3]. The discovery of microsatellite-instability high (MSI-H) as a biomarker for increased neoantigen burden and sensitivity to immune checkpoint blockage, for example, led to the design of a series of trials investigating Pembrolizumab in tumors harboring MSI-H regardless of organ of cancer origin [4, 5]. Promising results spanning several tumor types prompted a landmark decision by The U.S. Food and Drug Administration (FDA) in May 2017 by which Pembrolizumab was designated as the first tissue-agnostic cancer treatment [6, 7]. Trials used to develop Pembrolizumab as well as immune checkpoint inhibitors (ICI) Nivolumab and Atezolizumab relied on inclusive eligibility criteria facilitating the enrollment of many tumor types. Two drugs targeting tumors harboring neurotrophic tropomyosin receptor kinase gene fusions, received agnostic labels with accelerated approval by the FDA in 2018 (Larotrectinib) and 2019 (Entrectinib) [8].

Recent innovations in study design methodology with “master protocols” have extended clinical research beyond statistical estimation of the “population averaged” effects of a new drug for a single clinical indication, towards precision medicine [9–11]. Cancer trials devised to evaluate an intervention across multiple clinical indications with a shared feature, such as the same targeted genetic mutation, using a single protocol are often referred to as “basket trials” [12]. Cunanan, et al., review examples of high profile basket trials in oncology elucidating their flexibility [13]. Two notable, ongoing basket trials include the National Cancer Institute’s Molecular Analysis for Therapy Choice (NCI-MATCH) and the American Society of Clinical Oncology’s Targeted Agent and Profiling Utilization Registry (TAPUR). NCI-MATCH is an open phase II non-randomized basket trial with subprotocols by molecular subtype and matched treatment strategy for advanced solid tumors, lymphoma, and myeloma cancers. The trial’s design facilitates adaptive features such as adding or terminating individual baskets based on a given histology independently of the other ongoing baskets within the trial [14, 15]. TAPUR is also a phase II non-randomized trial enrolling patients with advanced cancers harboring potentially actionable genomic variants. Monitoring rules facilitate termination of enrollment to the study-specific drug-basket combinations in the absence of adequate efficacy, while allowing the remaining drug-basket combinations to continue enrollment. The trial has already identified the lack of sufficient clinical activity for sunitinib in patients with metastatic colorectal cancer and cetuximab in patients with advanced breast, non-small cell lung, or ovarian cancers without KRAS, NRAS, or BRAF mutations [16, 17].

Statistical frameworks for understanding and designing basket trials have been described in recent literature [18–28]. With recent widespread efforts to translate molecular-guided therapies into the clinic, these designs have become increasingly important [7, 21] and may require extension to facilitate seamless development strategies [29, 30]. The FDA and other national agencies are refining regulatory pathways for approving biomarker-driven histology-agnostic targeted and immunotherapeutic therapies. It is necessary that statistical evidence informing these decisions is acquired from appropriate statistical design with pre-specified rules for sequential monitoring. This article presents statistical and design considerations for basket trials based on existing frequentist and emerging Bayesian methodology. The designs are compared through simulation studies devised to evaluate potentially tumor agnostic therapies among multiple tumor histologies.

## 2. Statistical design considerations

Predominately used in phase II, basket trials are usually powered to detect a targeted level of clinical activity considered desirable for further development. Treatment success is typically defined by an objective response, which occurs with the presence of a partial or complete response following treatment as determined by RECIST [31] for solid tumors, or complete or partial remission for cancers of the blood [32]. Basket trials are often uncontrolled, with null response rates determined by statistical estimates reported in trials of appropriate chemotherapeutic agents currently used as standard of care regimens for the populations enrolled. Prospective trials of this type share common statistical considerations that are unique to basket trials. Trialists need to determine (a) how to control type I error while evaluating multiple tumor histologies and (b) how to analyze data across baskets. Given the choices of (a) and (b), one needs to determine how to make sequential decisions on the basis of accruing interim data over the course of the study. This section discusses statistical considerations confronting trialists implementing basket trials as well as the various options available on the basis of established frequentist and emerging Bayesian methodology.

### 2.1. Bayesian versus frequentist paradigms

Paradigms for statistical reasoning are defined by a dichotomy of frequentist versus Bayesian theory. The frequentist paradigm is founded by the perspective that probability is defined by the frequency with which a result occurs when a particular design is repeated ad infinitum. For frequentists, uncertainty is quantified in relation to asymptotic behavior. Statistical parameters are assumed to be fixed and unknown quantities. Hypothesis testing in the frequentist paradigm uses *p*-values which quantify the relative frequency of observing a statistic as, or more extreme than that observed in the experiment when one assumes a particular value for the null hypothesis. This conditional probability is conditioned on the particular experiment and null hypothesis. One should also note that hypothesis testing is intrinsically asymmetric, as failing to reject the null hypothesis does not imply support for an alternative. This feature of frequentist statistics gives rise to the adage, “the absence of evidence is not evidence of absence.”

By way of contrast, the Bayesian paradigm is founded on the perspective that probability should be defined in relation to one’s pre-existing belief. Statistical parameters assume “superpositions,” which are defined by probability distributions. Before initiating an experiment, Bayesians require that one translate their prior beliefs for statistical model parameters into *prior* distributions. Bayes rule is the mathematical theory that tells us how to combine the *prior* with data observed in an experiment to yield a *posterior* distribution, reflecting the synthesis of both prior belief and evidence. This posterior distribution may form the basis for a subsequent experiment where it plays the role as prior distribution. As such, the Bayesian paradigm provides the fundamental theory of sequential statistical learning from evidence. Unlike frequentist probability, Bayesian probability is not conditional on any experimental design. It rather depends on the prior distribution and data observed. Thus, statistical inference doesn’t depend on theoretical asymptotic behavior, but is rather defined entirely on the basis of the posterior distribution. This feature of Bayesian statistics enables decision-making on the basis of *posterior predictions* of future outcomes. In the context of clinical trials, for example, at any stage of the trial Bayesians can calculate the probability that the trial will conclude with a positive result given that it achieves planned enrollment. While previously limited by computational barriers, Bayesian methods have become increasingly accessible. Recent advances have demonstrated its advantages in efficiency when applied to clinical trials [33–36].

### 2.2. Decision making and type I error control

Decision making differs fundamentally between the Bayesian and frequentist paradigms. Frequentist decisions are rooted in hypothesis testing. Having assumed a null hypothesis, which for basket trials is represented by an objective response rate that is too low to justify further study, two types of errors may occur. A type I error occurs when the trial data satisfy the criteria to reject the null hypothesis when the null is actually true. Conversely, a type II error occurs when the therapy is truly efficacious, but the trial data fail to reject the null hypothesis. Relying on this framework, frequentists make decisions by applying *p*-value thresholds (e.g. *p*<0.05). *P*-value thresholds are calibrated, often under asymptotic assumptions, to control type I error rates at the specified threshold.

By way of contrast, model parameters exist not as fixed values, but as distributions, under the Bayesian paradigm. A null hypothesis is not required for statistical inference, which rather occurs with respect to the posterior distribution. That is to say, posterior distributions are conditional on the observed data and prior. Moreover, posterior inference does not require the abstraction of asymptotic re-sampling, but rather conforms more naturally to human conceptions of probability given the data observed. For example, using mathematical notation, *Pr*(*π*>0.10|*Y*) is the probability that objective response rate *π* exceeds 0.10 after having observed the data *Y*

The Bayesian paradigm provides two approaches to decision making. Posterior decisions occur by applying thresholds to posterior probabilities. For example, one may make the decision that a trial yielded promising results if *Pr*(*π*>0.10|*Y*)>0.95, that is, that the posterior probability that objective response rate *π* exceeds 0.10 is greater than 0.95 after observing the trial data *Y*. This form of “posterior” decision making is conditional on the data, *Y*, observed so far in an experiment. While not technically required, the concept of controlling type I error is necessary when designing an experiment [37]. Therefore, Bayesian designs should be calibrated to control type I and type II errors at acceptable levels. For posterior decisions, this requires selecting the posterior threshold, between 0 and 1, to control type I error at the desired level while minimizing type II errors for a given effect size. In practice this is done through simulation assuming fixed values of the targeted model parameters in order to estimate the frequentist operating characteristics of the type I and II error rates. For example, if one wishes to control the type I error rate at 10% for a design using Bayesian methods, 1,000 hypothetical null trials might be simulated with the estimated posterior probability calculated from each simulated trial. Then, the posterior probability threshold would be identified as the value where 10% of simulated null trials would result in a type I error (i.e., false positive conclusion). In practice, the statistical power of this posterior probability threshold would then be evaluated via simulation of non-null scenarios.

As noted above, predictions of future outcomes Y* yet unobserved arise seamlessly under the Bayesian paradigm. In fact, a full probability distribution for any collection of future responses is defined from its posterior predictive distribution (see Technical Appendix in S1 File for detailed derivation). Thus, having observed interim data from a partially enrolled trial, the Bayesian paradigm provides the probability that the trial would conclude successfully if the trial continues to full enrollment. Posterior predictive decisions require two thresholds. A posterior probability threshold applied for decision making after full enrollment is achieved as well as a predictive threshold applied to the probability that the trial eventually yields a successful conclusion (as defined by the posterior threshold) given partial enrollment. Facilitating highly flexible sequential designs and design adaptations, this aspect of decision making is unique to the Bayesian paradigm. It does require the calibration of type I and type II errors over a matrix of both thresholds, which is challenging for trialists in the absence of readily available software. Recent open-source tools have filled this gap, however. See, for example, the R software package “ppseq,” which provides tools to design a clinical trial with sequential predictive probability monitoring (http://www.emilyzabor.com/ppseq) [38]. In practice, the approach of calibrating the posterior predictive probability threshold to terminate for futility is analogous to the approach described for calibrating the posterior probability threshold described previously, however a posterior probability threshold must already be selected and may either be based on a model without interim monitoring or with an iterative process as implemented in the “ppseq” package [38].

#### 2.2.1. Types of type I error control

There are two types of frequentist type I errors that arise with basket trials: basket-wise and family-wise. The basket-wise type I error rate, also called the marginal type I error rate in some sources, describes the rate at which a type I error occurs for an individual basket. Basket trials may have multiple indications for which the response rate of the targeted therapy under study is too low to consider further development. The family-wise type I error rate considers all null baskets conjointly. It represents the false positive rate for *at least one* of the null baskets, reflecting more stringent control [18, 19]. Both frequentist hypothesis testing and Bayesian posterior and predictive decision making can be calibrated to control family-wise and basket-wise errors. In the frequentist context, this reflects the conclusions of a hypothesis test that may be based on a p-value (e.g., p<0.05), whereas in the Bayesian context this would be calculated after setting thresholds (e.g., a posterior probability > 0.95). The type I error rates can then be estimated via simulation study, where the proportion of times null basket(s) are incorrectly declared efficacious (i.e., p<0.05 or posterior probability > 0.95) would represent the type I error rate.

### 2.3. Sequential decision making

Sequential designs allow for decisions to be made during the course of conducting a trial from accruing data at interim analyses. Sequential analysis introduces multiple comparisons which inflate the chance that a type I error may occur.

#### 2.3.1. Frequentist approaches

Frequentists make sequential decisions from interim data by identifying appropriate *p*-value thresholds for each planned interim evaluation. The set of thresholds are selected to control type I and type II error rates. Often referred to as “alpha-spending,” the thresholds typically vary across evaluation periods as a function of an information metric defining the extent of information that has accrued in relation to the design’s planned sample size (or planned number of events for time-to-event models). Thresholds are chosen collectively to preserve the overall type I error rate (rate at which a false positive is made at any interim analysis) at a pre-specified level while “spending” parts of it among various interim evaluations [39]. Typically, less alpha spending occurs with less follow-up so that early evaluations must achieve more extreme result in order to halt the trial [40]. The lack of a consistent boundary for all evaluations may also be considered a limitation relative to designs with a constant boundary. Moreover, the likelihood of early stopping can be more or less aggressive depending on the type of alpha-spending methodology used.

The predominate frequentist approach used in trials with a binary endpoint, such as many basket trials, is the Simon two-stage design [41]. This design allows for a single interim analysis with the option to stop the trial for futility if fewer than some set number of successes are observed. If the study does not terminate for futility, it continues to the maximum sample size. The final conclusion as to whether to reject or fail to reject the null hypothesis is also mapped to a pre-determined number of observed successes. Simon originally proposed two approaches for designing two-stage trials. The minimax design is calibrated to minimize the maximum sample size, while the optimal design minimizes the expected sample size.

Many basket trials used Simon’s two-stage designs calibrated to control basket-wise type I error rates independently among a collection of enrolled tumor histologies. Vemurafenib was studied for patients with *BRAF*^*V*600*E*^ mutation–positive cancers using this approach. Baskets were included for non-small-cell lung cancer (NSCLC), cholangiocarcinoma, Erdheim-Chester disease or Langerhans’-cell histiocytosis (ECD/LCH), anaplastic thyroid cancer, breast cancer, ovarian cancer, multiple myeloma, colorectal cancer, and a ninth basket for all other eligible cancer types [2]. Preliminary efficacy was seen in the NSCLC and ECD/LCH baskets, whereas three baskets (breast cancer, ovarian cancer, and multiple myeloma) did not have sufficient enrollment for the initial futility evaluation. The SUMMIT trial evaluated Neratinib treatment in patients with *HER*2− and *HER*3−mutant cancers comprising lung, breast, bladder, colorectal, biliary tract, endometrial, cervical, gastroesophageal, ovarian, and ‘other’ [3]. Baskets were analyzed independently using the optimal Simon’s two-stage design. Initial results supported the activity of Neratinib only in the breast cancer cohort, with several baskets failing to reach enrollment targets by the time of interim analysis. These studies highlight some of the limitations of applying independent Simon’s two-stage designs independently for each basket including: the failure to formally identify futility or efficacy in baskets with low enrollment, low power within individual baskets, and lack of control of family-wise type I error rates inherent to multiple hypothesis testing.

As demonstrated with the real-world examples, the simplicity of Simon’s two-stage design leads to some trade-offs that warrant further consideration. First, there is only one interim analysis. In cases where there is no signal of efficacy, it may be possible to terminate the study earlier using alternative approaches to interim monitoring. Second, in the context of basket trials, the implementation of Simon’s two-stage design assumes each basket is independent and it will fail to synthesize evidence across baskets in the presence of consistent results. Sharing information across exchangeable baskets could further improve the ability to discern signals for both futility and efficacy, as well as address potential sample size imbalances across baskets that are present in many trials. It is these two limitations of this popular frequentist study design that motivate this examination of novel basket trial designs that apply Bayesian methods for interim monitoring and also have the potential to share information across baskets when appropriate.

#### 2.3.2. Bayesian approaches

The Bayesian paradigm offers the unique advantage of conducting sequential trials with decisions at interim analyses based on prediction. While Bayesian methods for sequential analysis can be based on either posterior or predictive probability [22, 34, 42–45], predictive probability designs synthesize the uncertainty yet to be observed, which allows for consistent decision thresholds across all interim analyses. Predictive probabilities computed at interim analyses quantify the risk of continuing study enrollment. In practice, predictive probability thresholds closer to 0 lead to less frequent stopping for futility, whereas values near 1 would stop in the absence of an almost certain success (e.g. all enrolled patients respond). The optimal choice for the predictive probability threshold depends upon evaluation of trial operating characteristics via simulation study to ensure the desired type I error rate is met while achieving adequate statistical power.

**Fig 1:**
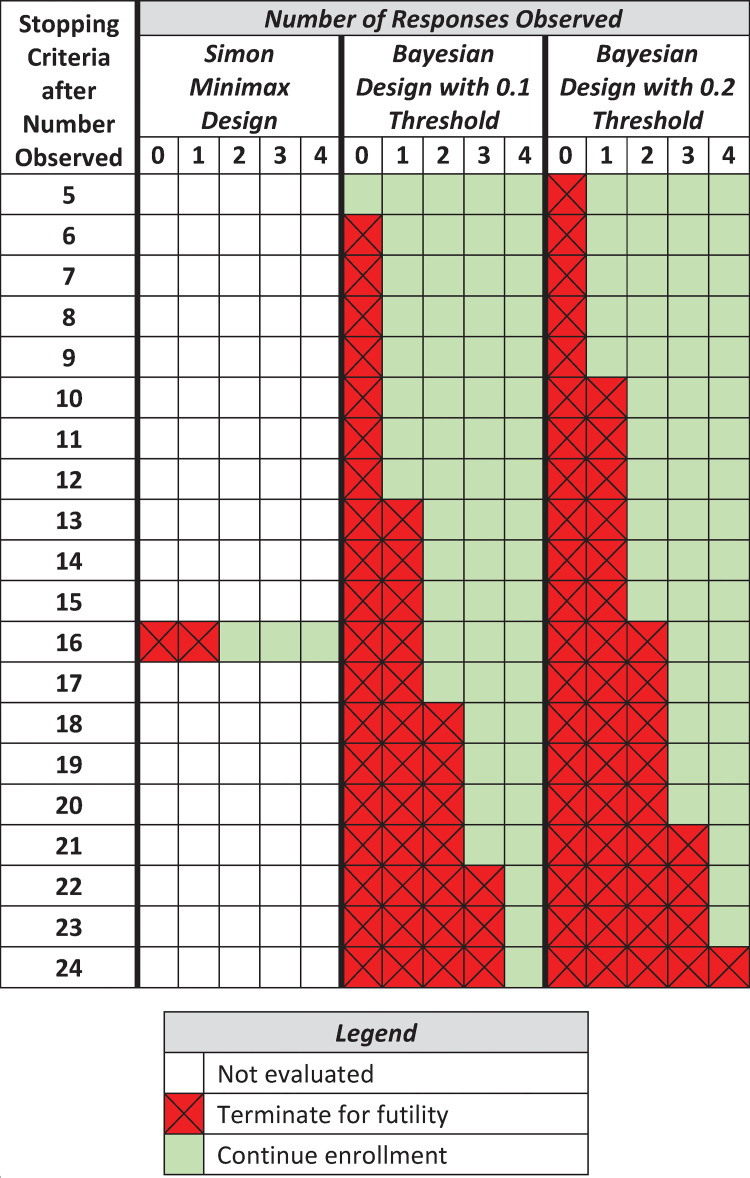
Comparison of sequential designs for a one-arm trial to compare a null response rate of 10% and a target response rate of 30% to achieve 90% power and a 10% type I error rate under Simon’s two-stage minimax design, which enrolls a maximum sample size of 25. Simon’s two-stage design assumes a single interim analysis after 16 participant outcomes are observed and would terminate for futility only if 0 or 1 response was observed. On the other hand, Bayesian designs with predictive probability monitoring may be more flexible. A 0.1 and 0.2 threshold are illustrated, where an arm could terminate for futility if no responses are observed within the first 6 participants for a threshold of 0.1 or 5 participants for a threshold of 0.2. Further, the Bayesian designs facilitate a continual evaluation of futility over the course of the study, with larger thresholds more aggressively terminating early for futility (e.g., after 10 participants are observed, the 0.1 threshold terminates if no responses are observed, whereas the 0.2 threshold terminates if 1 or fewer responses are observed.

This approach can be used to increase the frequency of interim analyses beyond the single evaluation facilitated by the Simon two-stage design. Fig 1 illustrates the decision rules resulting from the ubiquitous Simon two-stage design versus Bayesian design with predictive probability monitoring for a single clinical indication. Decision rules were calibrated for a null response rate of 10% and a target response rate of 30% with 90% power and 10% type I error rate control. For Simon’s minimax design, this results in a maximum sample size of 25 with a single interim analysis for futility after 16 participants have completed the study. Color is used in Fig 1 to depict the occurrence of an interim evaluation. Red indicates the recommendation to terminate the trial, whereas green indicates continued enrollment. The Simon design requires 16 participants before any decision can be made about a given clinical indication. The design stops a basket for futility if fewer than 2 objective responses are observed among the first 16 patients.

Bayesian predictive probability design enables continual interim monitoring after each observation, starting after the fifth patient. Fig 1 presents two examples with 0.1 and 0.2 predictive probability thresholds. Therefore, at any evaluation time the trial’s predictive probability of eventual success (with complete enrollment) must exceed 0.1 or 0.2, respectively, to continue accrual to a given basket. With the 0.1 threshold, a basket could terminate as soon as 6 total participants are observed in the absence of a single objective response. Interestingly, the 0.1 threshold recommends the same stopping criteria as Simon’s two-stage minimax design after 16 participants have been observed. The Bayesian design with 0.2 predictive probability threshold is more aggressive in that it will more often stop for futility than lower thresholds.

### 2.4. Information sharing across baskets

Basket trials are designed with the intrinsic assumption that patients with different tumor types (or clinical indications) that harbor a common actionable molecular feature will respond to the target therapy. Yet designs predominately fail to formally share information across the collection of indications using an appropriate statistical model.

Assuming the absence of heterogeneity among tumor types, some trials have pooled data across baskets, such as pembrolizumab for several solid tumors with mismatch-repair deficiency [4]. Very few therapies (three at this time) have demonstrated tumor agnostic efficacy, making this a strong and likely incorrect assumption. Moreover, basket trials often fail to enroll equitably across tumor types. For example, the SUMMIT trial enrolled across 10 baskets with a minimum of 4 to a maximum of 26 patients in each basket [3]. In the presence of treatment effect heterogeneity, pooling data acquired from multiple baskets results in highly biased statistical estimates that are dominated by the tumor type(s) that happen to enroll more patients, but fail to describe the individual baskets. Moreover, this approach fails to characterize the evidence for a tumor agnostic label.

At the other extreme, many trials treat each basket independently, which explicitly accounts for the potential for heterogeneity, but does not allow for direct quantification of treatment effect heterogeneity. Moreover, with imbalanced enrollment, which is commonplace, independent analyses limit statistical power to reach a meaningful conclusion for small baskets (e.g. less than 16 patients as per the Simon design shown in Fig 1). This approach, however, benefits from simplicity using traditional statistical methods for clinical trials based on frequentist hypothesis testing.

The Bayesian paradigm offers another avenue for analysis of basket trials using hierarchical models devised to share information among the tumor types enrolled. This allows trialists to explicitly estimate the extent of heterogeneity evident from the trial data (integrating both small and large baskets without pooling) while quantifying an overall decision regarding the evidence for a tumor agnostic label. Several statistical methods have been developed to facilitate information sharing among potentially “non-exchangeable” data sources [46–50]. A general class of models, referred to as multi-source exchangeability models (MEMs), have been developed specifically for basket trials with sequential analysis [22, 51]. MEMs effectively integrate information among baskets by modeling all possible “pooling” relationships among a collection of tumor types. The analysis strategy enables the identification of meta-baskets with tumor agnostic effects. Additionally, Simon, et al., proposed a Bayesian hierarchical modeling strategy that facilitates information sharing across baskets based on a prior probability that all baskets are correlated and the prior probability that a drug is active in any specific basket [50]. Modeling details are provided in the Supplementary Materials in S1 File for these methods.

## 3. Design comparison

This section uses simulation to compare statistical designs of basket trials implemented with the Simon two-stage design [41] and three different Bayesian designs: interim monitoring for futility with posterior probability and information sharing using Simon’s 2016 approach [50], interim monitoring for futility with predictive probability but not information sharing, and interim monitoring for futility with predictive probability and information sharing using multi-source exchangeability models [22, 51].

### 3.1. Simulation design

Statistical design operating characteristics are computed from 1000 replicate simulated trials each enrolling *N* = 25 patients across 10 baskets. The objective response rate for each basket is assumed to be either 10% (the null value) or 30% (targeted alternative response rate). The sample size of 25 per basket was identified from the power calculation resulting from calculations corresponding to a Simon two-stage minimax design. All three Bayesian designs implement continual interim monitoring for each basket after the 5th participant. The impact of predictive probability threshold was evaluated across a grid from 0 (i.e., no interim monitoring) to 0.5 (i.e., more aggressive interim stopping for futility) in increments of 0.05.

Two types of scenarios are presented. Global scenarios reflect the absence of heterogeneity. All baskets assume either the null or alternative response rate. A mixed scenario is also presented for which 8 baskets are null and 2 are alternative. The global scenario reflects a setting that is optimal for information sharing since all baskets are truly statistically exchangeable. The mixed scenario reflects the reality of tumor heterogeneity and limited effectiveness. The performance of each method is summarized by several statistical measures. Basket-wise and family-wise type I error rates are calculated for each scenario. The expected sample size summarizes the average number of participants that are enrolled in a basket. The stopping rate for early termination due to futility is presented within each basket along with the probability that all null baskets terminate early for futility. All simulations were implemented in R v4.0.1 (Vienna, Austria). Additional details pertaining to model calibration are presented in the Supplementary Materials in S1 File with statistical derivations.

### 3.2. Results

**Fig 2:**
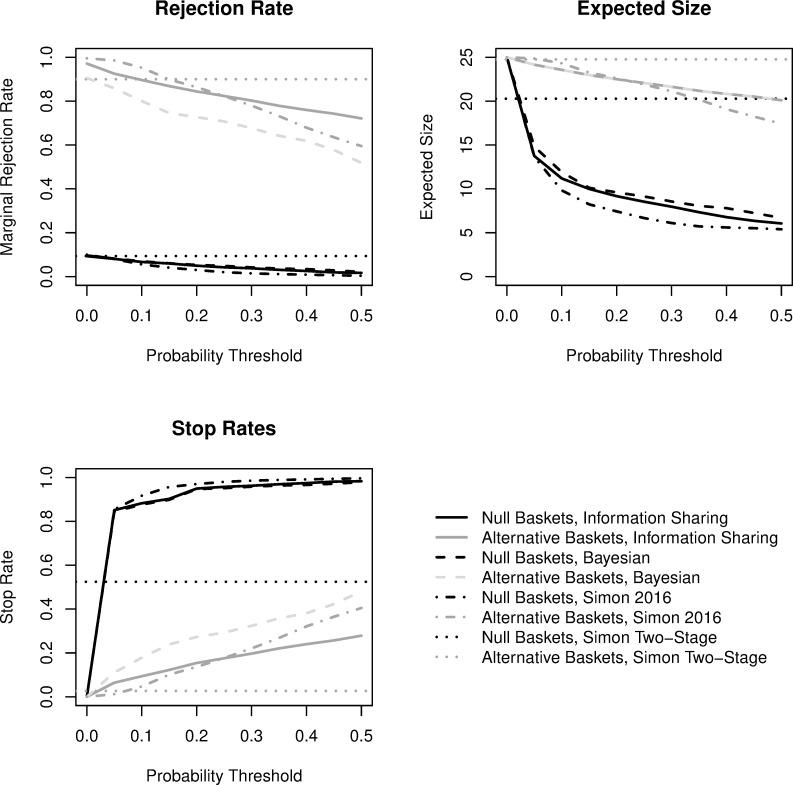
Comparisons of trial operating characteristics for *global* scenarios (all null or all alternative baskets). Black coloring is for null baskets and gray coloring for alternative baskets. The dotted lines represent Simon’s two-stage minimax design, dashed lines represent a design with interim monitoring after each participant based on Bayesian predictive probability futility monitoring, dashed-dotted lines represent Simon’s (2016) design with information sharing with posterior probability futility monitoring, and the solid lines represent a Bayesian design that also facilitates information sharing across baskets based on exchangeability of the response rate with predictive probability futility monitoring. The rejection rate summarizes the proportion of baskets across the 1000 simulated trials where efficacy was concluded, the expected sample size presents the average number enrolled in a given null or alternative basket, and the stop rate describes the proportion of baskets that terminated early at any point for futility.

Results for global scenarios are presented in Fig 2. Simon’s minimax design is depicted by horizontal lines while operating characteristics for the Bayesian predictive probability (PP) designs vary as a function of the PP threshold. Bayesian PP designs that share information across baskets are denoted by “Information Sharing.” Basket-wise type I error is shown here, while results for family-wise type I error are provided in the Supplementary Materials in S1 File. Simon’s minimax two-stage design achieves 90% power while controlling the basket-wise type I error rate at 9.3%.

For the Bayesian designs, both power and type I error decrease with increasing PP threshold. As expected based on the calibration of the designs, without information sharing, the PP design achieves equivalent power only with a PP threshold of 0. However, in the presence of information sharing, the PP design with threshold 0.05 attains both larger power (92.5%) with a lower type I error rate (8.1%) when compared to Simon’s two-stage design. This improvement is even larger for Simon’s posterior probability design, where it attains a power of 98.5% with a 0.05 posterior probability threshold. Increasing the PP threshold to 0.1 results in power of 89.6% (similar to the Simon design) while nearly halving the type I error rate to 6.7%. This performance is also seen with Simon’s posterior probability design where a posterior probability threshold of 0.1 results in 95.2% power with a lower 5.6% type I error rate. As the thresholds continue to increase beyond 0.1, the designs with information sharing have decreasing power as a trade-off for a further decreasing type I error. In the global scenarios, with information sharing it is possible to increase the power while decreasing the type I error rate using PP thresholds of 0.05 or 0.1 when compared to Simon’s two-stage minimax design.

The expected basket sample size for Simon’s two-stage design is 20.3 under the global null scenario and 24.8 under the global alternative scenario. All Bayesian designs show an improvement in the expected sample size under the global null scenario even with the conservative 0.05 threshold. The PP designs achieve an expected sample size of only 14.8 without information sharing and 13.8 with information sharing under the null scenario, while Simon’s posterior probability design achieves an expected sample size of 13.6. These are all approximately a 30% decrease when compared to Simon’s two-stage minimax design. Under the global alternative scenario the expected sample sizes are 23.7, 24.2, and 24.9, respectively, for the designs without and with information sharing and Simon’s posterior probability design using a threshold of 0.05. This is demonstrated visually by the early stopping rates in Fig 2. The stopping rate for alternative baskets under all Bayesian designs is greater than Simon’s two-stage design. Interestingly, as noted previously, with information sharing there is greater power than the Simon two-stage design even in the presence of higher stopping rate for alternative baskets with lower thresholds.

Tables presented in the Supplementary Materials in S1 File include additional summaries worth noting for the global scenarios. For all designs, the family-wise type I error rate across all 10 baskets is drastically inflated beyond 10%. This is not surprising given the calibration to target a 10% basket-wise type I error rate. The probability of all 10 null baskets stopping in a given trial ranges from 24.2% with a PP threshold of 0.05 for the Bayesian design with information sharing to 85.5% with a PP threshold of 0.50. For Simon’s posterior probability design, this ranges from 31.3% at a posterior probability threshold of 0.05 to 96.3% with a threshold at 0.5. Simon’s two-stage design only has a 0.1% probability of all 10 null baskets terminating, suggesting that the implementation of frequent interim monitoring using PP protects against falsely positive conclusions overall.

**Fig 3:**
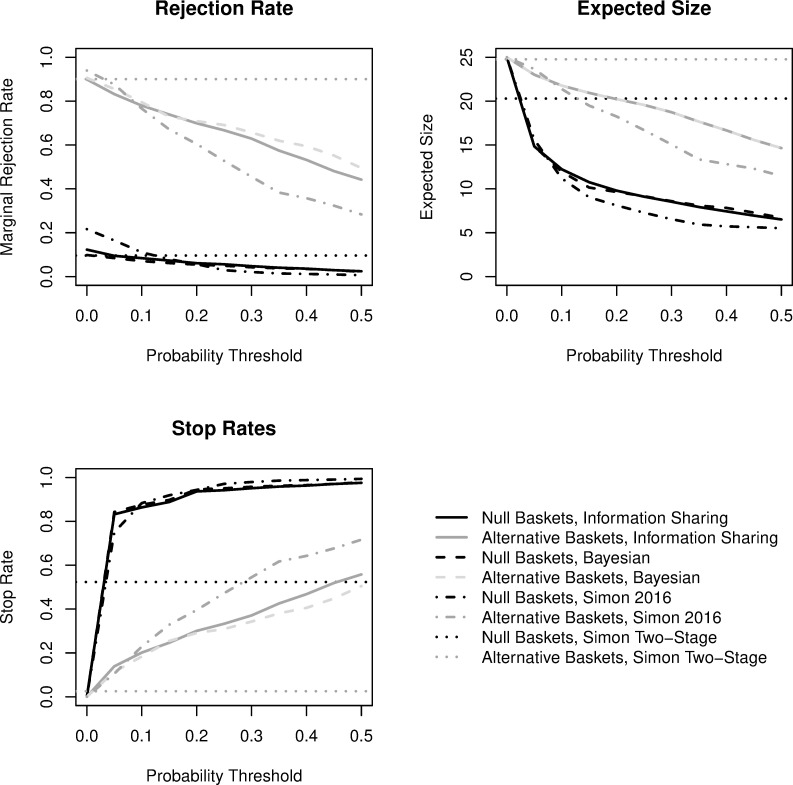
Comparisons of trial operating characteristics for *mixed* scenario (8 null, 2 alternative baskets). Black coloring is for null baskets and gray coloring for alternative baskets. The dotted lines represent Simon’s two-stage minimax design, dashed lines represent a design with interim monitoring after each participant based on Bayesian predictive probability futility monitoring, dashed-dotted lines represent Simon’s (2016) design with information sharing with posterior probability futility monitoring, and the solid lines represent a Bayesian design that also facilitates information sharing across baskets based on exchangeability of the response rate with predictive probability futility monitoring. The rejection rate summarizes the proportion of baskets across the 1000 simulated trials where efficacy was concluded, the expected sample size presents the average number enrolled in a given null or alternative basket, and the stop rate describes the proportion of baskets that terminated early at any point for futility.

Results for the mixed scenario simulated with 8 null and 2 alternative baskets are presented in Fig 3. Because Simon’s design and the Bayesian design without information sharing evaluate each basket independently, trends are similar to those observed in Fig 2. With two alternative baskets, the results between Bayesian PP designs (with and without information sharing) are more similar at lower PP thresholds. A slight reduction in power is observed at higher PP thresholds (e.g., 44.2% with information sharing at a PP threshold of 0.50 versus 49.5% without information sharing). These results also demonstrate that even when calibrated for fixed sample designs, at lower PP thresholds Bayesian designs achieve gains in trial efficiency with respect to the expected sample size (and stopping rates) of null baskets. Simon’s posterior probability design has a type I error rate of 21.6% without interim monitoring, and rates of 16.5% and 11.1% for thresholds of 0.05 and 0.1, respectively.

**Fig 4:**
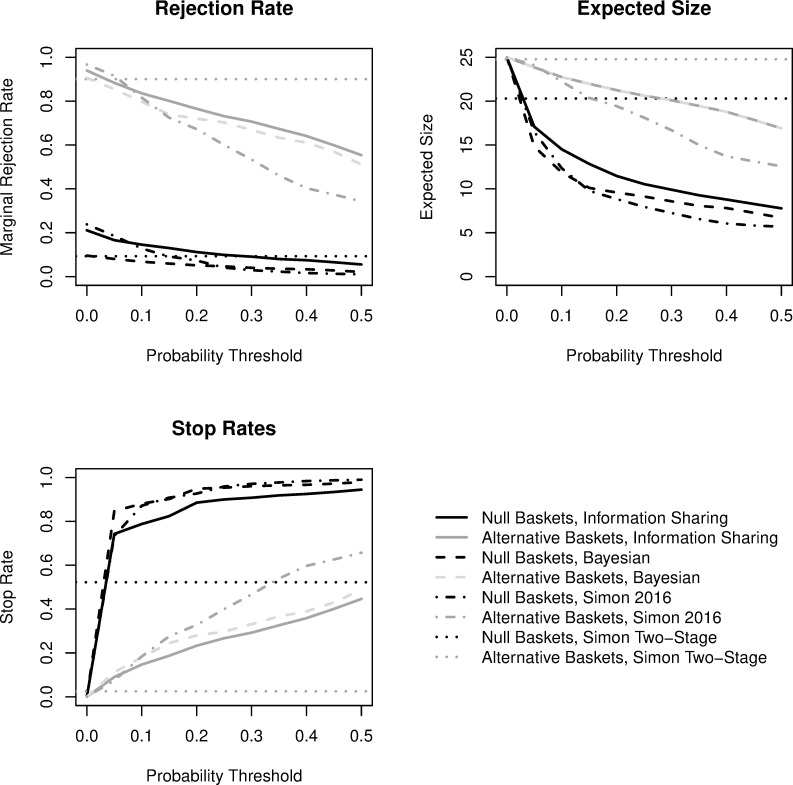
Comparisons of trial operating characteristics for *equally mixed* scenario (5 null, 5 alternative baskets). Black coloring is for null baskets and gray coloring for alternative baskets. The dotted lines represent Simon’s two-stage minimax design, dashed lines represent a design with interim monitoring after each participant based on Bayesian predictive probability futility monitoring, dashed-dotted lines represent Simon’s (2016) design with information sharing with posterior probability futility monitoring, and the solid lines represent a Bayesian design that also facilitates information sharing across baskets based on exchangeability of the response rate with predictive probability futility monitoring. The rejection rate summarizes the proportion of baskets across the 1000 simulated trials where efficacy was concluded, the expected sample size presents the average number enrolled in a given null or alternative basket, and the stop rate describes the proportion of baskets that terminated early at any point for futility.

When considering an equally mixed scenario with 5 null and 5 alternative baskets (Fig 4), there are very similar responses to the mixed scenario with 8 null and 2 alternative baskets. One exception is the Bayesian PP design with information sharing is more similar to Simon’s posterior probability design, where the type I error rate at low thresholds is elevated above 10%. For designs without information sharing, since each basket is evaluated independently, we observe similar trial operating characteristics as the other scenarios.

## 4. Discussion

While offering new treatment pathways for patients living with refractory cancers, advances in molecularly targeted therapies pose challenges to the traditional processes for experimental design. Design innovations have emerged with master protocols, providing frameworks for designing trials for precision medicine, such as basket trials. This article discussed the fundamental statistical considerations for basket trial design from both frequentist and Bayesian perspectives. Our findings suggest that methodology for basket trials could be improved further through the implementation of Bayesian design.

Bayesian design with continual predictive probability monitoring (after every participant), while more complex than Simon’s two-stage design, demonstrated clear advantages for intermediate-phased basket trials devised with interim decision making using low PP thresholds. Further, Simon’s posterior probability design for futility monitoring that also facilitates information sharing provides improvements over the frequentist Simon two-stage design. Bayesian design with information sharing demonstrated advantages for global scenarios, resulting in increased power and decreased type I error rates relative to Simon’s two-stage design. For mixed scenarios the Bayesian designs with information sharing performed similarly to the design without information sharing, which is encouraging given these scenarios are more challenging for methods that facilitate information sharing. This suggests that under the scenarios explored, it is advantageous to incorporate information sharing given the minimal trade-offs observed across the simulated trials.

There are limitations to consider with our proposed design and the included simulation studies. First, we calibrated the posterior probability thresholds to evaluate efficacy for the Bayesian designs assuming the global scenario without interim analyses. One should note that calibration for scenarios with interim monitoring or under the mixed scenario would produce different results. Detailed discussions of issues and solutions to design calibration for trials that study multiple indications can be found [18, 19]. A second consideration is that we assumed constant accrual across tumor types. Many basket trials conclude with imbalanced enrollment among the study clinical indications. Hobbs and Landin [22] interrogated the impact of imbalanced enrollment based on interim analysis of the Vemurafenib basket trial. While not explicitly considered in this article, we can note that the potential impact of imbalance on the proposed Bayesian design is mitigated with information sharing. Zhou and Ji [52] presented an approach for basket trials with information sharing based on hierarchical models while also incorporating a formal robust Bayesian hypothesis testing framework, providing a meaningful alternative to examining the posterior credible intervals for statistical inference.

Clinical trials are devised for specific contexts. The relative importance of statistical operating characteristics must be weighed in consideration of practicality. Relying on a single futility interim evaluation for each basket, Simon’s two-stage design benefits from simplicity. While more complex, Bayesian predictive probability designs offer advantages that compound with additional tumor types. To avoid interim computation, it is possible to conduct Bayesian designs using decision tables (similar to Fig 1) computed in advance of the trial’s initiation. To facilitate seamless implementation of the Bayesian methods discussed in this article we have made the code used for simulation studies available on GitHub (https://github.com/alexbiostats/Sequential-PP-Design) and also have produced the “basket” package in R to implement the multi-source exchangeability model framework for basket trials or other related master protocol settings [20, 53]. These innovations in monitoring and information sharing can be embedded within other types of master protocols to yield more efficient designs.

## Supporting information

S1 File Supplementary materials. Greater statistical model details and background on multi-source exchangeability models are included in the Supplementary Materials. Additional simulation results, tables, and figures are presented in the Supplementary Materials file.
(PDF)
