# Supplementary material for: Bayesian and frequentist approaches to sequential monitoring for futility in oncology basket trials: A comparison of Simon’s two-stage design and Bayesian predictive probability monitoring with information sharing across baskets
Source: PLoS One. 2022 Aug 2;17(8):e0272367. doi: 10.1371/journal.pone.0272367 (PMC9345361; doi:10.1371/journal.pone.0272367)
Supplement: S1 File — Greater statistical model details and background on multi-source exchangeability models are included in the Supplementary Materials. Additional simulation results, tables, and figures are presented in the Supplementary Materials file. (PDF) [file pone.0272367.s001.pdf]

Supplementary materials for

Understanding basket trials from Bayesian and Frequentist perspectives

Alexander M. Kaizer, Emily C. Zabor, Lei Nie, Brian P. Hobbs

1 Statistical Details

1.1 Multisource Exchangeability Models

Multi-source exchangeability models (MEMs) are a general Bayesian framework based upon Bayesian model averaging that were initially proposed for an "asymmetric" setting where there is one primary source of data and supplemental sources from other studies [1, 2]. However, in a basket trial, we have a "symmetric" setting where each basket can be considered a primary source and all other baskets are supplemental sources within the same study. Methods to account for this symmetric setting were extended from the asymmetric setting by Hobbs and Landin, which we describe briefly below [3].

MEMs define all possible combinations of assuming pairwise exchangeability between each basket in the basket trial with the other $J-1$ baskets, resulting in 2^{J-1} combinations of exchangeability for each basket with respect to all other baskets. Each of these configurations of exchangeability represent a multi-source exchangeability model. In the MEM approach, a prior weight is derived for each configuration of exchangeability by specifying the prior with respect to the source (i.e., basket) rather than the each model as is done in Bayesian model averaging [1, 3]. This results in a reduction in the dimensionality of the prior specification space for each basket from 2^{J-1} to $J-1$. Priors on source exchangeability have been developed for both empirically and fully Bayesian

approaches [2]. In this paper we focus on an empirically Bayesian prior that is detailed in Hobbs and Landin, which identifies the maximum *a posteriori* (MAP) multi-source exchangeability model when evaluating all baskets simultaneously [3]. However, using the single MAP MEM results in all posterior inference based on one model that does not account for the uncertainty in our estimation of exchangeability in a finite sample. To induce exploration of subconfigurations of exchangeability, a constraint hyperparameter $B \in (0, 1)$ is introduced. A value of $B = 1$ represents the MAP configuration, $B = 0$ represents a model with no sharing of information across baskets, and intermediate values average over the possible model configurations between these extremes.

In the context of the presented simulations, $B = 0$ was selected to represent a Bayesian model with predictive probability monitoring for futility without information sharing. For MEMs where information sharing is implemented, the hyperparameter $B = 0.1$ is selected based on prior work that demonstrates a more conservative approach to information sharing that still induces improvements in trial operating characteristics [2, 4, 5]. In practice, higher hyperparameter values for B to allow more liberal information sharing or fully Bayesian priors of MEMs can be implemented and calibrated to achieve the desired type I error rates.

Posterior inference with MEMs is based upon the weighted average from all posterior model weights calculated from Bayesian model averaging. Similarly, the application of predictive probability monitoring at an interim stage of the trial as described in the Technical Appendix of the main manuscript can be applied to the posterior averaged across all MEMs. In our implementation the posterior weights for exchangeability of each configuration are estimated from the interim data, then 5000 possible future sets of results are simulated with the future posterior probability calculated with the same posterior weights for each MEM applied to calculate the PP. As in the Technical Appendix, if the PP is less than the selected threshold, ϕ, the basket would terminate for futility and cease enrollment, whereas other baskets can continue enrolling.

Greater details about the general structure of MEMs and the asymmetric implementation can be found in Section 2 and 3 for Gaussian outcomes of Kaizer, Koopemeiners, and Hobbs [1]. For binary outcomes additional details can be found in Kaizer, Hobbs, and Koopmeiners on the general structure in Sections 3.1 and 3.2 and prior specification considerations in Section 3.3 [2]. For more

specific details on the symmetric context, Section 2.2 of the Hobbs and Landin paper introduces the general MEMs specification, Section 2.3 discusses how to estimate posterior probabilities and the effective sample size, and Section 2.4 presents the application of MEMs in the context of basket trials and the estimation of basketwise exchangeability [3].

1.2 Simon's Basket Trial Design with Information Sharing

Simon, et al., proposed a basket trial design for binary outcomes that could implement futility monitoring via posterior probabilities instead of the predictive probability while facilitating information sharing across baskets through a Bayesian hierarchical model [6]. One advantage of using posterior probabilities is that it can be more computationally efficient than calculating predictive probabilities. However, this comes at the expense of not accounting for the uncertainty of the future trial enrollment and what may be concluded if the trial reached full enrollment. We briefly describe the general approach of Simon below, but note that the have an interactive online Shiny app and have made R code available via GitHub.

Briefly, they propose a model with two hypotheses with corresponding priors specified: (1) the probability all baskets are homogeneous (i.e., exchangeable) and (2) the probability of activity in any given basket. In their original work, examples included a prior probability of homogeneous response of 0.5 and any basket having activity of 0.33. However, these priors can be set based on a given context. We detail our specific assumptions in the simulation design section below.

1.3 Bayesian Posterior Predictive Probability Calculations

This section demonstrates in detail Bayesian posterior predictive probability models used to formulate decision rules for monitoring futility during enrollment, and deciding whether sufficient "improvement" is evident given that the maximum sample size is achieved. This is presented in the absence of information sharing, where closed form solutions are available.

For basket trial with J baskets ($j = 1, ..., J$), let $Y_{i,j} = 1$ if the ith participant in the jth basket experiences a success and $Y_{i,j} = 0$ if the treatment fails to meet the efficacy outcome. π_j represents the response rate for the jth basket. The posterior for π_j with a Beta(α_j, β_j) prior after

observing r_j responses from n_j patients follows the beta distribution

$$f(\pi_j \mid r_j) = \frac{1}{\beta(r_j + a_j,\, n_j - r_j + b_j)} \pi_j^{r_j + a_j - 1} (1 - \pi_j)^{n_j - r_j + b_j - 1},$$

where $\beta()$ represent the beta function and $0 \leq \pi_j \leq 1$.

Let N_{max} denote the planned maximum total number of patients that may enroll into each basket. Then N_j denotes a random variable counting the total number of patients enrolled among N_{max} in the jth basket. Let R_j denote a random variable counting the number of responders among N_{max} patients treated with the jth therapy. The predictive probability of observing s_j successes (responses) among $N_{max} - n_j$ future patients can be expressed as a product of gamma functions,

$$\begin{aligned} Pr(R_j = r_j + s_j \mid r_j) = &\frac{\Gamma(N_{max} - n_j + 1)}{\Gamma(s_j + 1)\Gamma(N_{max} - n_j - s_j + 1)} \\ &\times \frac{\Gamma(s_j + r_j + a_j)\Gamma(N_{max} - s_j - r_j + b_j)}{\Gamma(N_{max} + a_j + b_j)} \times \frac{\Gamma(n_j + a_j + b_j)}{\Gamma(r_j + a_j)\Gamma(n_j - r_j + b_j)}, \end{aligned}$$

where $s_j = 0, \ldots, N_{max} - n_j$.

Mathematically, the binary decision pertaining to whether sufficient improvement was evident is an evaluation of the posterior probability

$$Pr(\pi_j > \pi_0 \mid R_j) > \theta,$$

where π_0 represents the assumed response rate under the null. The posterior threshold $\theta \in (0, 1)$ controls the amount of "evidence" required to conclude success. Using properties of the beta distribution, this posterior probability follows as

$$Pr(\pi_j > \pi_0 \;\Big|\; R_j) = 1 - \frac{\int_0^{\pi_0} u^{R_j + a_j - 1}(1 - u)^{N_{max} - R_j + b_j - 1} du}{\beta(R_j + a_j,\, N_{max} - R_j + b_j)}.$$

In the presence of partial enrollment, $n_j < N_{max}$, the predictive probability (PP) that the trial

ultimately demonstrates improvement for treatment in basket j follows as

$$\begin{aligned}\lambda(r_j) &= E_{R_j}[I\{\, Pr(\pi_j > \pi_0 \mid R_j) > \theta \,\}] \\ &= \sum_{v=0}^{N_{max}-n_j} I\{\, Pr(\pi_j > \pi_0 \mid r_j + v) > \theta \,\} \\ &\qquad \times Pr(R_j = r_j + v \mid r_j),\end{aligned}$$

where $I\{\}$ represents the indicator function. The decision to terminate enrollment in the jth basket after observing n_j patients follows from evaluating the PP of eventual success

$$\begin{aligned}&\lambda(r_j) < \phi, \text{terminate enrollment to the } j^{th} \text{ basket for futility,} \\ &\lambda(r_j) \geq \phi, \text{continue enrolling patients to the } j^{th} \text{ basket,}\end{aligned}$$

for a given threshold $\phi \in (0, 1)$.

2 Simulation Study Details

2.1 Simulation Design

In our simulation studies we assume the null response rate is $p_0 = 0.1$ and the target response rate is $p_1 = 0.3$ for a basket trial enrolling across 10 baskets. Equal accrual is assumed across baskets throughout the duration of the simulated trials. A maximum sample size of 25 was selected to achieve 90% power and a 10% basket-wise type I error rate for Simon's minimax two-stage design [7] with a comparison to two Bayesian designs with predictive probability (PP) monitoring that are described below.

Simon's minimax two-stage design within each basket enrolls a maximum sample size of 25 with the only interim analysis after 16 participants. If 0 or 1 responses to treatment are observed at the interim analysis, the basket would terminate for futility. Otherwise, if the basket continues enrolling to a maximum of 25 participants, the decision to recommend further studies occurs if 5 or more responses are observed.

We consider three Bayesian designs in our simulation study: one without information sharing (i.e., $B = 0$), one with some information sharing using MEMs with $B = 0.1$, and an approach by Simon, et al., implementing information sharing through a hierarchical model [6]. Details on the priors and hyperparameter choices are given in the following paragraphs.

For the Bayesian designs without information sharing (i.e., $B = 0$) and with information sharing using MEMs (i.e., $B = 0.1$), a $(0.5, 0.5)$ prior is placed on the response. The selection of $B = 0.1$ is based on prior studies where it has performed well with respect to the trade-off of sharing information while still offering improvements in power or reduced type I error rates in certain scenarios of interest, however we note more generally that hyperparameters such as values of B should be calibrated to each context in practice [5].

For Simon's design with posterior probability monitoring for futility, a prior on homogeneous response is set at 0.1 to reflect the hyperparameter assumed for MEMs. The prior for any basket showing efficacy is set at 0.33 based on their original paper.

Interim monitoring for futility is commenced after the 5th participant is observed and continues after each additional participant until the basket terminates for futility or the maximum sample size of 25 is enrolled. For designs with information sharing, terminated baskets are still considered in the evaluation of exchangeability even though they are no longer enrolling as they still contribute valuable information on treatment response. In all Bayesian designs the effect of PP thresholds is examined across a range of values from 0 to 0.5 by increments of 0.05. This reflects a range without stopping for futility (i.e., a fixed sample design with a PP threshold of 0) to thresholds with more aggressive termination for futility.

2.2 Simulation Results

2.2.1 Posterior Probability Calibration

The posterior probability for efficacy is calibrated to achieve a 10% basket-wise type I error rate in the global null scenario without interim monitoring futility: 0.154 for Simon's Bayesian design, 0.900 for $B = 0$, 0.848 for $B = 0.1$. Calibration that maintains a 10% family-wise type I error rate across all baskets was also explored: 0.665 for Simon's Bayesian design, 0.990 for $B = 0$, and 0.979

for $B = 0.1$.

Calibration in all cases was based on simulating 1,000 fixed sample trials for the global null (all 10 baskets with a 10% response) and estimating the posterior probabilities for each basket in the simulated trial. Then, the threshold was identified so that a type I error rate of 10% for a given setting (basket-wise or family-wise control) was maintained. The calculated threshold was used for the mixed scenarios corresponding to their respective basket-wise or family-wise simulations.

2.2.2 Simon Two-Stage Design Simulation Results

Table 1 summarizes the results for the Simon two-stage approach across the three simulation scenarios. It is important to note that the Simon design is not calibrated to maintain a family-wise type I error rate, so only the basket-wise type I error rate is controlled (i.e., the columns labeled "Rejection Rate").

	Null Baskets					*Alternative Baskets*		
Scenario	Rejection Rate	Family-wise Rate	Expected Size	Stop Rate	Prob. All Null Stop	Rejection Rate	Expected Size	Stop Rate
Global	0.093	0.626	20.3	0.524	0.001	0.900	24.8	0.027
Mixed 8/2	0.096	0.558	20.3	0.523	0.005	0.900	24.8	0.025
Mixed 5/5	0.093	0.384	20.3	0.522	0.000	0.900	24.8	0.025

Table 1: Simon design for the global and both mixed scenarios. The "Mixed 8/2" scenario includes 8 null and 2 alternative baskets. The "Mixed 5/5" scenario includes 5 null and 5 alternative baskets.

2.2.3 Basket-wise (Marginal) Type I Error Rate Control Simulation Results

This section presents the tabular results presented in Figures 1 and 2 of the manuscript, along with additional information for the family-wise type I error rate and the probability that all null baskets stopped early for futility.

	Null Baskets										*Alternative Baskets*					
PP Threshold	Rejection Rate		Family-wise Rate		Expected Size		Stop Rate		Prob. All Null Stop		Rejection Rate		Expected Size		Stop Rate	
	B=0	B=0.1	B=0	B=0.1	B=0	B=0.1	B=0	B=0.1	B=0	B=0.1	B=0	B=0.1	B=0	B=0.1	B=0	B=0.1
0	0.096	0.093	0.635	0.559	25.0	25.0	0.000	0.000	0.000	0.000	0.905	0.971	25.0	25.0	0.000	0.000
0.05	0.082	0.081	0.563	0.543	14.8	13.8	0.844	0.851	0.211	0.242	0.859	0.925	23.7	24.2	0.111	0.064
0.1	0.070	0.067	0.508	0.478	11.9	11.2	0.877	0.882	0.282	0.320	0.801	0.896	22.3	23.6	0.178	0.094
0.15	0.061	0.060	0.453	0.442	10.1	10.0	0.898	0.902	0.355	0.393	0.746	0.869	21.0	23.0	0.239	0.122
0.2	0.053	0.050	0.418	0.386	9.6	9.2	0.947	0.950	0.582	0.614	0.727	0.844	20.7	22.5	0.273	0.154
0.25	0.049	0.042	0.391	0.340	9.1	8.5	0.951	0.957	0.609	0.660	0.707	0.824	20.3	22.1	0.293	0.176
0.3	0.042	0.038	0.348	0.295	8.6	8.0	0.958	0.962	0.652	0.705	0.676	0.802	19.7	21.6	0.324	0.197
0.35	0.037	0.031	0.312	0.247	8.1	7.3	0.963	0.969	0.688	0.753	0.642	0.779	19.1	21.2	0.358	0.221
0.4	0.034	0.025	0.297	0.210	7.8	6.8	0.966	0.975	0.703	0.790	0.619	0.760	18.6	20.8	0.381	0.240
0.45	0.028	0.019	0.253	0.166	7.3	6.4	0.972	0.981	0.747	0.834	0.577	0.743	17.7	20.5	0.423	0.257
0.5	0.021	0.017	0.198	0.145	6.7	6.1	0.979	0.983	0.802	0.855	0.518	0.721	16.4	20.1	0.482	0.279

Table 2: Global scenario, basket-wise type I error rate calibration across various predictive probability (PP) thresholds. $B = 0$ represents the Bayesian design without information sharing, $B = 0.1$ represents the design with information sharing through MEMs.

	Null Baskets					*Alternative Baskets*		
PP Threshold	Rejection Rate	Family-wise Rate	Expected Size	Stop Rate	Prob. All Null Stop	Rejection Rate	Expected Size	Stop Rate
0	0.100	0.602	25.0	0.000	0.000	0.996	25.0	0.000
0.05	0.080	0.512	13.6	0.855	0.313	0.985	24.9	0.012
0.1	0.056	0.393	9.8	0.917	0.515	0.952	24.3	0.047
0.15	0.041	0.302	8.2	0.957	0.698	0.899	23.3	0.101
0.2	0.030	0.243	7.4	0.970	0.757	0.864	22.6	0.136
0.25	0.020	0.173	6.7	0.980	0.827	0.823	21.9	0.177
0.3	0.014	0.132	6.1	0.986	0.868	0.779	21.1	0.221
0.35	0.011	0.103	5.7	0.989	0.897	0.732	20.2	0.268
0.4	0.009	0.081	5.6	0.991	0.919	0.679	19.1	0.321
0.45	0.006	0.055	5.5	0.994	0.945	0.635	18.2	0.365
0.5	0.004	0.037	5.4	0.996	0.963	0.595	17.4	0.405

Table 3: Global scenario, basket-wise type I error rate calibration across various predictive probability (PP) thresholds for Simon's 2016 design.

	Null Baskets										*Alternative Baskets*					
PP Thres hold	Rejection Rate		Family-wise Rate		Expected Size		Stop Rate		Prob. All Null Stop		Rejection Rate		Expected Size		Stop Rate	
	B=0	B=0.1	B=0	B=0.1	B=0	B=0.1	B=0	B=0.1	B=0	B=0.1	B=0	B=0.1	B=0	B=0.1	B=0	B=0.1
0	0.098	0.123	0.565	0.559	25.0	25.0	0.000	0.000	0.000	0.000	0.905	0.899	25.0	25.0	0.000	0.000
0.05	0.084	0.095	0.497	0.492	14.9	14.8	0.843	0.832	0.266	0.284	0.856	0.832	23.7	23.0	0.114	0.139
0.1	0.071	0.084	0.438	0.438	11.9	12.2	0.876	0.864	0.357	0.372	0.795	0.779	22.2	21.8	0.183	0.202
0.15	0.062	0.073	0.387	0.396	10.1	10.8	0.898	0.888	0.437	0.439	0.730	0.739	20.7	20.9	0.254	0.246
0.2	0.054	0.061	0.355	0.346	9.6	9.8	0.946	0.936	0.645	0.650	0.709	0.699	20.4	20.2	0.291	0.300
0.25	0.049	0.056	0.330	0.321	9.2	9.1	0.951	0.942	0.670	0.675	0.691	0.667	20.1	19.6	0.309	0.333
0.3	0.042	0.048	0.290	0.274	8.6	8.5	0.958	0.951	0.710	0.722	0.657	0.629	19.4	18.7	0.343	0.371
0.35	0.037	0.040	0.262	0.237	8.1	7.9	0.963	0.959	0.738	0.761	0.620	0.575	18.7	17.7	0.380	0.425
0.4	0.035	0.036	0.247	0.210	7.8	7.4	0.965	0.963	0.753	0.789	0.595	0.532	18.3	16.7	0.406	0.468
0.45	0.029	0.029	0.213	0.172	7.3	7.0	0.971	0.970	0.787	0.828	0.552	0.481	17.3	15.6	0.448	0.519
0.5	0.022	0.024	0.163	0.147	6.7	6.5	0.978	0.976	0.837	0.853	0.495	0.442	16.0	14.6	0.505	0.558

Table 4: Mixed scenario (8 null, 2 alternative), basket-wise type I error rate calibration across various predictive probability (PP) thresholds. $B = 0$ represents the Bayesian design without information sharing, $B = 0.1$ represents the design with information sharing through MEMs.

	Null Baskets					*Alternative Baskets*		
PP Threshold	Rejection Rate	Family-wise Rate	Expected Size	Stop Rate	Prob. All Null Stop	Rejection Rate	Expected Size	Stop Rate
0	0.216	0.819	25.0	0.000	0.000	0.940	25.0	0.000
0.05	0.165	0.722	15.6	0.751	0.121	0.876	23.7	0.105
0.1	0.111	0.565	11.2	0.883	0.398	0.766	21.4	0.229
0.15	0.079	0.462	9.0	0.919	0.529	0.671	19.5	0.329
0.2	0.057	0.351	8.1	0.943	0.649	0.605	18.2	0.396
0.25	0.029	0.204	7.3	0.971	0.796	0.527	16.7	0.472
0.3	0.021	0.152	6.5	0.979	0.848	0.455	15.1	0.545
0.35	0.014	0.105	5.9	0.986	0.895	0.383	13.4	0.617
0.4	0.012	0.089	5.7	0.988	0.911	0.357	12.8	0.642
0.45	0.009	0.071	5.6	0.991	0.929	0.324	12.3	0.676
0.5	0.006	0.049	5.5	0.994	0.951	0.283	11.5	0.717

Table 5: Mixed scenario (8 null, 2 alternative), basket-wise type I error rate calibration across various predictive probability (PP) thresholds for Simon's 2016 design.

	Null Baskets										*Alternative Baskets*					
PP Thres hold	Rejection Rate		Family-wise Rate		Expected Size		Stop Rate		Prob. All Null Stop		Rejection Rate		Expected Size		Stop Rate	
	B=0	B=0.1	B=0	B=0.1	B=0	B=0.1	B=0	B=0.1	B=0	B=0.1	B=0	B=0.1	B=0	B=0.1	B=0	B=0.1
0	0.096	0.211	0.393	0.649	25.0	25.0	0.000	0.000	0.000	0.000	0.906	0.940	25.0	25.0	0.000	0.000
0.05	0.080	0.166	0.335	0.535	14.8	17.1	0.847	0.743	0.447	0.312	0.855	0.883	23.7	23.8	0.112	0.090
0.1	0.068	0.146	0.293	0.491	11.9	14.5	0.880	0.788	0.530	0.392	0.797	0.835	22.3	22.7	0.180	0.147
0.15	0.059	0.130	0.261	0.449	10.1	12.8	0.902	0.824	0.598	0.453	0.739	0.801	20.9	22.0	0.245	0.187
0.2	0.051	0.112	0.231	0.404	9.6	11.5	0.949	0.885	0.769	0.585	0.720	0.765	20.6	21.2	0.280	0.233
0.25	0.047	0.099	0.212	0.370	9.1	10.5	0.953	0.900	0.788	0.625	0.703	0.731	20.2	20.6	0.297	0.267
0.3	0.040	0.091	0.186	0.348	8.6	9.9	0.960	0.908	0.814	0.647	0.669	0.707	19.6	20.1	0.331	0.292
0.35	0.036	0.080	0.168	0.313	8.1	9.3	0.964	0.918	0.832	0.680	0.633	0.673	18.9	19.5	0.367	0.326
0.4	0.033	0.075	0.158	0.294	7.8	8.8	0.967	0.925	0.842	0.704	0.611	0.641	18.5	18.8	0.389	0.359
0.45	0.028	0.065	0.135	0.257	7.3	8.3	0.972	0.934	0.865	0.740	0.570	0.599	17.5	17.9	0.430	0.401
0.5	0.021	0.055	0.103	0.219	6.7	7.8	0.979	0.945	0.897	0.780	0.511	0.554	16.2	16.9	0.489	0.446

Table 6: Equally mixed scenario (5 null, 5 alternative), basket-wise type I error rate calibration across various predictive probability (PP) thresholds. $B = 0$ represents the Bayesian design without information sharing, $B = 0.1$ represents the design with information sharing through MEMs.

	Null Baskets					*Alternative Baskets*		
PP Threshold	Rejection Rate	Family-wise Rate	Expected Size	Stop Rate	Prob. All Null Stop	Rejection Rate	Expected Size	Stop Rate
0	0.238	0.756	25.0	0.000	0.000	0.967	25.0	0.000
0.05	0.185	0.633	16.5	0.734	0.222	0.916	24.1	0.074
0.1	0.128	0.484	12.3	0.871	0.516	0.816	22.2	0.184
0.15	0.092	0.360	9.8	0.908	0.640	0.724	20.4	0.276
0.2	0.073	0.292	8.8	0.927	0.708	0.672	19.4	0.328
0.25	0.041	0.170	7.9	0.959	0.830	0.600	18.1	0.400
0.3	0.029	0.128	7.2	0.971	0.872	0.533	16.7	0.467
0.35	0.023	0.102	6.6	0.977	0.898	0.460	15.0	0.540
0.4	0.016	0.076	6.1	0.984	0.924	0.403	13.7	0.597
0.45	0.013	0.062	5.8	0.987	0.938	0.374	13.1	0.626
0.5	0.010	0.051	5.7	0.990	0.949	0.343	12.6	0.657

Table 7: Equally mixed scenario (5 null, 5 alternative), basket-wise type I error rate calibration across various predictive probability (PP) thresholds for Simon's 2016 design.

2.2.4 Family-wise Type I Error Control Simulation Results

Simulation results where the Bayesian design posterior probability thresholds were calibrated to maintain a 10% family-wise type I error rate are presented. The Bayesian designs show a decrease in statistical power given the more stringent criteria of family-wise type I error rate control, but the trade-off is a basket-wise type I error rate well below 10% across all thresholds and a nearly 100% stop rate for null baskets. Further, there is a greater than 82.1% probability of all null baskets stopping across all scenarios with interim monitoring with MEMs, however this is lower with Simon's 2016 design at lower predictive probability thresholds. This indicates that MEMs are more likely to stop all null baskets due to futility than other approaches, but this does come at the cost of lower statistical power than Simon's 2016 design.

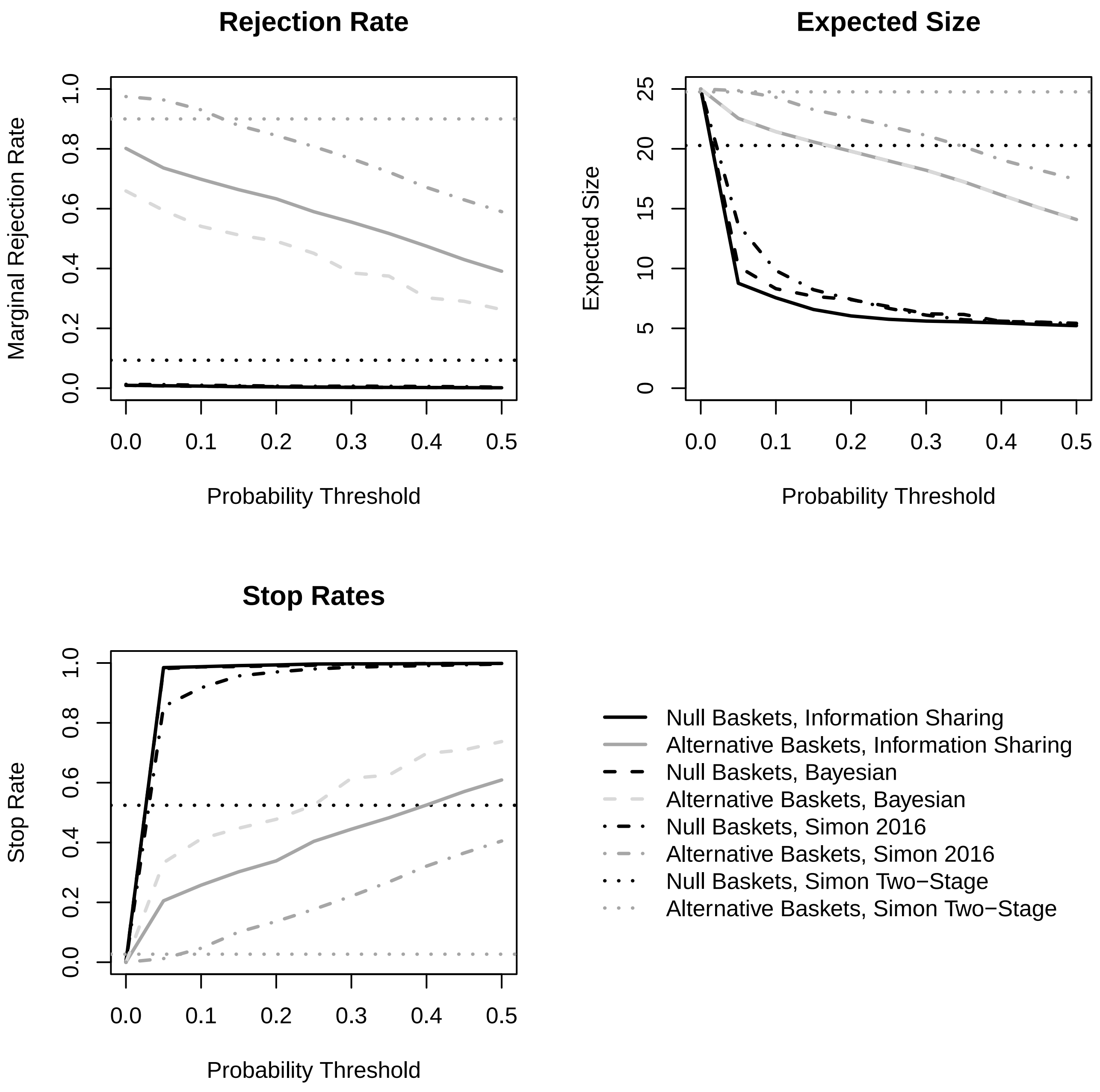

Figure 1: Summary of scenario results for **global** scenarios (all null or all alternative baskets), calibrated to maintain the *family-wise* type I error without interim monitoring. Black coloring is for null baskets and gray coloring for alternative baskets. TThe dotted lines represent Simon's two-stage minimax design, dashed lines represent a design with interim monitoring after each participant based on Bayesian predictive probability futility monitoring, dashed-dotted lines represent Simon's (2016) design with information sharing with posterior probability futility monitoring, and the solid lines represent a Bayesian design that also facilitates information sharing across baskets based on exchangeability of the response rate with predictive probability futility monitoring. The rejection rate summarizes the proportion of baskets across the 1000 simulated trials where efficacy was concluded, the expected sample size presents the average number enrolled in a given null or alternative basket, and the stop rate describes the proportion of baskets that terminated early at any point for futility.

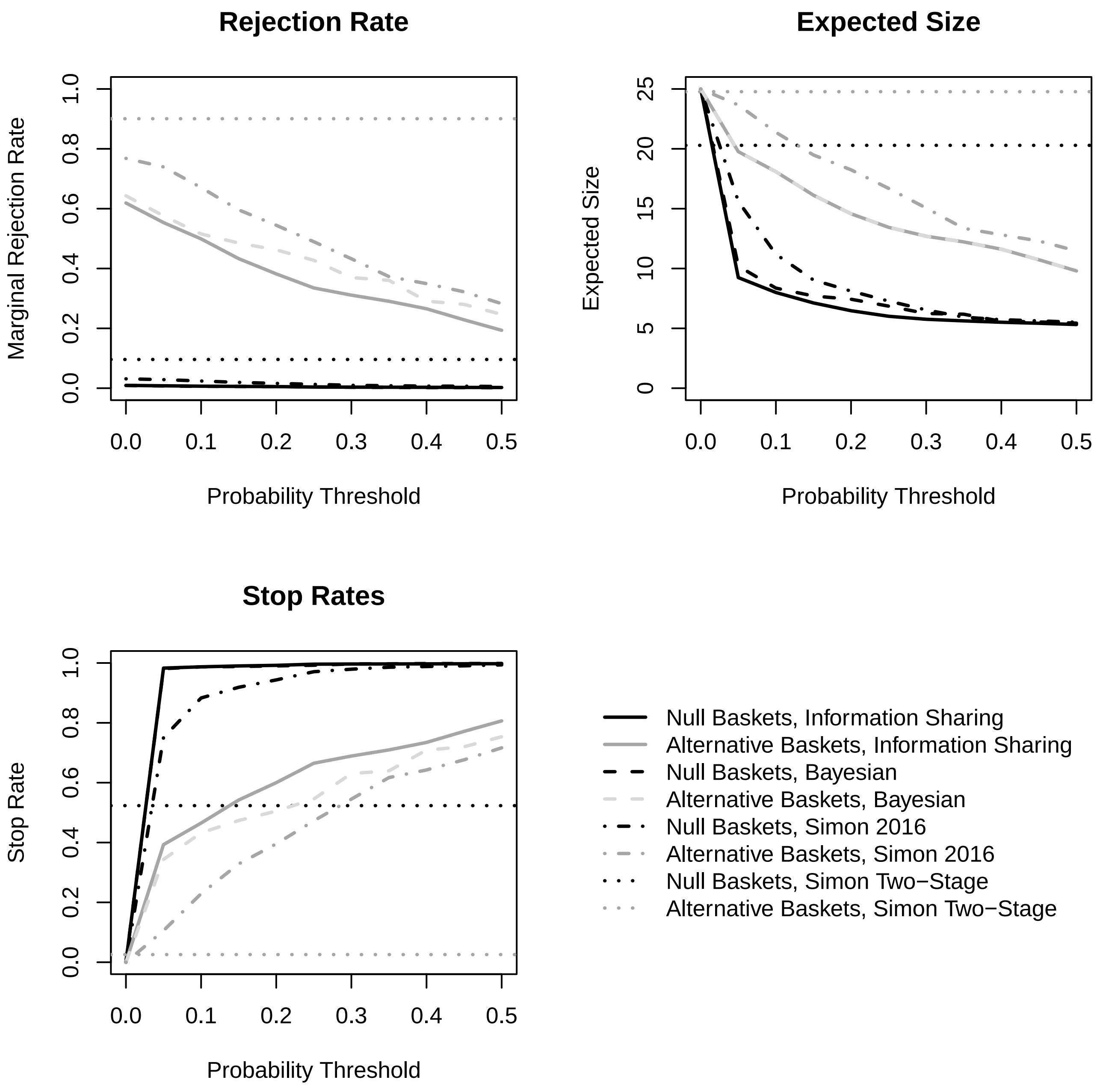

Figure 2: Summary of scenario results for **mixed** scenario (8 null, 2 alternative baskets), calibrated to maintain the *family-wise* type I error without interim monitoring. Black coloring is for null baskets and gray coloring for alternative baskets. The dotted lines represent Simon's two-stage minimax design, dashed lines represent a design with interim monitoring after each participant based on Bayesian predictive probability futility monitoring, dashed-dotted lines represent Simon's (2016) design with information sharing with posterior probability futility monitoring, and the solid lines represent a Bayesian design that also facilitates information sharing across baskets based on exchangeability of the response rate with predictive probability futility monitoring. The rejection rate summarizes the proportion of baskets across the 1000 simulated trials where efficacy was concluded, the expected sample size presents the average number enrolled in a given null or alternative basket, and the stop rate describes the proportion of baskets that terminated early at any point for futility.

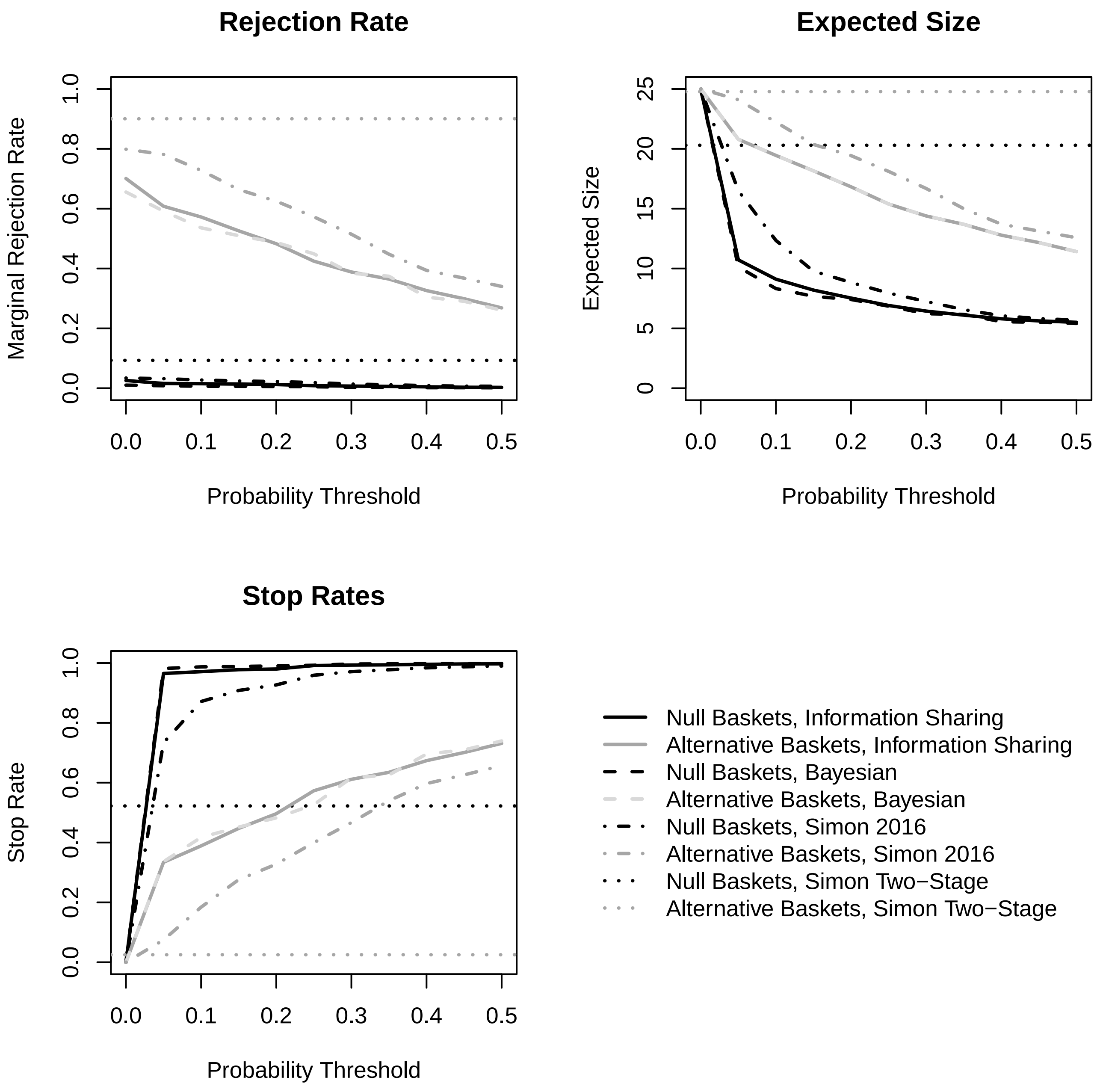

Figure 3: Summary of scenario results for **equally mixed** scenario (5 null, 5 alternative baskets), calibrated to maintain the *family-wise* type I error without interim monitoring. Black coloring is for null baskets and gray coloring for alternative baskets. The dotted lines represent Simon's two-stage minimax design, dashed lines represent a design with interim monitoring after each participant based on Bayesian predictive probability futility monitoring, dashed-dotted lines represent Simon's (2016) design with information sharing with posterior probability futility monitoring, and the solid lines represent a Bayesian design that also facilitates information sharing across baskets based on exchangeability of the response rate with predictive probability futility monitoring. The rejection rate summarizes the proportion of baskets across the 1000 simulated trials where efficacy was concluded, the expected sample size presents the average number enrolled in a given null or alternative basket, and the stop rate describes the proportion of baskets that terminated early at any point for futility.

	Null Baskets										*Alternative Baskets*					
PP Thres hold	Rejection Rate		Family-wise Rate		Expected Size		Stop Rate		Prob. All Null Stop		Rejection Rate		Expected Size		Stop Rate	
	B=0	B=0.1	B=0	B=0.1	B=0	B=0.1	B=0	B=0.1	B=0	B=0.1	B=0	B=0.1	B=0	B=0.1	B=0	B=0.1
0	0.010	0.010	0.095	0.093	25.0	25.0	0.000	0.000	0.000	0.000	0.659	0.801	25.0	25.0	0.000	0.000
0.05	0.008	0.008	0.075	0.080	10.2	8.8	0.981	0.985	0.821	0.854	0.594	0.736	21.1	22.5	0.333	0.205
0.1	0.006	0.007	0.063	0.068	8.3	7.6	0.987	0.988	0.873	0.880	0.541	0.698	19.3	21.4	0.412	0.258
0.15	0.006	0.005	0.059	0.049	7.7	6.6	0.988	0.991	0.884	0.914	0.512	0.663	18.4	20.6	0.448	0.302
0.2	0.005	0.004	0.051	0.043	7.4	6.0	0.990	0.994	0.905	0.936	0.491	0.633	17.9	19.8	0.478	0.339
0.25	0.004	0.003	0.041	0.033	6.8	5.8	0.993	0.997	0.927	0.966	0.451	0.590	16.7	19.0	0.525	0.404
0.3	0.003	0.003	0.032	0.029	6.2	5.6	0.997	0.997	0.968	0.971	0.385	0.555	15.1	18.2	0.615	0.445
0.35	0.003	0.003	0.029	0.027	6.2	5.5	0.997	0.997	0.971	0.973	0.375	0.517	14.8	17.2	0.625	0.483
0.4	0.002	0.002	0.021	0.023	5.6	5.5	0.998	0.998	0.979	0.977	0.302	0.475	12.6	16.1	0.698	0.525
0.45	0.002	0.002	0.019	0.018	5.5	5.3	0.998	0.998	0.981	0.982	0.290	0.430	12.4	15.1	0.710	0.570
0.5	0.002	0.002	0.015	0.015	5.4	5.2	0.999	0.999	0.985	0.985	0.263	0.391	11.7	14.1	0.737	0.609

Table 8: Global scenario, family-wise type I error rate calibration across various predictive probability (PP) thresholds. $B = 0$ represents the Bayesian design without information sharing, $B = 0.1$ represents the design with information sharing through MEMs.

	Null Baskets					*Alternative Baskets*		
PP Threshold	Rejection Rate	Family-wise Rate	Expected Size	Stop Rate	Prob. All Null Stop	Rejection Rate	Expected Size	Stop Rate
0	0.013	0.100	25.0	0.000	0.000	0.975	25.0	0.000
0.05	0.012	0.097	13.6	0.855	0.313	0.963	24.9	0.012
0.1	0.010	0.081	9.8	0.917	0.515	0.930	24.3	0.047
0.15	0.009	0.073	8.2	0.957	0.698	0.878	23.3	0.101
0.2	0.007	0.063	7.4	0.970	0.757	0.845	22.6	0.136
0.25	0.006	0.057	6.7	0.980	0.827	0.807	21.9	0.177
0.3	0.007	0.067	6.1	0.986	0.868	0.767	21.1	0.221
0.35	0.006	0.062	5.7	0.989	0.897	0.722	20.2	0.268
0.4	0.006	0.056	5.6	0.991	0.919	0.671	19.1	0.321
0.45	0.005	0.048	5.5	0.994	0.945	0.630	18.2	0.365
0.5	0.004	0.034	5.4	0.996	0.963	0.590	17.4	0.405

Table 9: Global scenario, family-wise type I error rate calibration across various predictive probability (PP) thresholds for Simon's 2016 design.

	Null Baskets										*Alternative Baskets*					
PP Thres hold	Rejection Rate		Family-wise Rate		Expected Size		Stop Rate		Prob. All Null Stop		Rejection Rate		Expected Size		Stop Rate	
	B=0	B=0.1	B=0	B=0.1	B=0	B=0.1	B=0	B=0.1	B=0	B=0.1	B=0	B=0.1	B=0	B=0.1	B=0	B=0.1
0	0.009	0.009	0.072	0.070	25.0	25.0	0.000	0.000	0.000	0.000	0.643	0.619	25.0	25.0	0.000	0.000
0.05	0.007	0.008	0.059	0.062	10.2	9.2	0.981	0.983	0.854	0.871	0.576	0.553	21.0	19.8	0.344	0.393
0.1	0.006	0.007	0.049	0.053	8.4	8.0	0.987	0.987	0.899	0.901	0.515	0.499	19.0	18.1	0.432	0.465
0.15	0.006	0.006	0.047	0.046	7.7	7.1	0.988	0.990	0.909	0.924	0.485	0.433	17.9	16.1	0.473	0.542
0.2	0.005	0.005	0.041	0.041	7.4	6.5	0.990	0.992	0.924	0.940	0.463	0.382	17.4	14.6	0.505	0.600
0.25	0.004	0.004	0.034	0.029	6.9	6.0	0.993	0.996	0.941	0.970	0.427	0.335	16.3	13.4	0.545	0.665
0.3	0.003	0.004	0.026	0.027	6.2	5.8	0.997	0.997	0.974	0.973	0.369	0.311	14.7	12.7	0.630	0.689
0.35	0.003	0.003	0.023	0.025	6.2	5.6	0.997	0.997	0.977	0.975	0.360	0.290	14.5	12.2	0.639	0.710
0.4	0.002	0.003	0.016	0.023	5.6	5.5	0.998	0.997	0.984	0.977	0.291	0.266	12.3	11.6	0.709	0.735
0.45	0.002	0.003	0.016	0.020	5.5	5.4	0.998	0.997	0.984	0.980	0.280	0.229	12.1	10.7	0.720	0.771
0.5	0.002	0.002	0.013	0.018	5.4	5.3	0.998	0.998	0.987	0.982	0.246	0.194	11.4	9.8	0.753	0.806

Table 10: Mixed scenario (8 null, 2 alternative), family-wise type I error rate calibration across various predictive probability (PP) thresholds. $B = 0$ represents the Bayesian design without information sharing, $B = 0.1$ represents the design with information sharing through MEMs.

	Null Baskets					*Alternative Baskets*		
PP Threshold	Rejection Rate	Family-wise Rate	Expected Size	Stop Rate	Prob. All Null Stop	Rejection Rate	Expected Size	Stop Rate
0	0.031	0.229	25.0	0.000	0.000	0.768	25.0	0.000
0.05	0.029	0.212	15.6	0.751	0.121	0.740	23.7	0.105
0.1	0.024	0.180	11.2	0.883	0.398	0.670	21.4	0.229
0.15	0.019	0.148	9.0	0.919	0.529	0.596	19.5	0.329
0.2	0.016	0.122	8.1	0.943	0.649	0.544	18.2	0.396
0.25	0.013	0.096	7.3	0.971	0.796	0.489	16.7	0.472
0.3	0.010	0.072	6.5	0.979	0.848	0.432	15.1	0.545
0.35	0.008	0.062	5.9	0.986	0.895	0.373	13.4	0.617
0.4	0.007	0.054	5.7	0.988	0.911	0.349	12.8	0.642
0.45	0.007	0.051	5.6	0.991	0.929	0.322	12.3	0.676
0.5	0.005	0.043	5.5	0.994	0.951	0.282	11.5	0.717

Table 11: Mixed scenario (8 null, 2 alternative), family-wise type I error rate calibration across various predictive probability (PP) thresholds for Simon's 2016 design.

	Null Baskets										*Alternative Baskets*					
PP	Rejection		Family-wise		Expected		Stop		Prob. All		Rejection		Expected		Stop	
Thres	Rate		Rate		Size		Rate		Null Stop		Rate		Size		Rate	
hold	B=0	B=0.1	B=0	B=0.1	B=0	B=0.1	B=0	B=0.1	B=0	B=0.1	B=0	B=0.1	B=0	B=0.1	B=0	B=0.1
0	0.010	0.026	0.051	0.124	25.0	25.0	0.000	0.000	0.000	0.000	0.656	0.701	25.0	25.0	0.000	0.000
0.05	0.008	0.016	0.041	0.077	10.1	10.7	0.982	0.965	0.908	0.844	0.592	0.608	21.1	20.8	0.336	0.334
0.1	0.007	0.015	0.033	0.074	8.3	9.1	0.987	0.971	0.935	0.868	0.536	0.572	19.2	19.4	0.417	0.389
0.15	0.006	0.014	0.032	0.068	7.7	8.2	0.988	0.978	0.942	0.897	0.510	0.526	18.3	18.2	0.452	0.447
0.2	0.006	0.012	0.028	0.060	7.4	7.5	0.990	0.980	0.951	0.905	0.487	0.483	17.7	16.8	0.482	0.496
0.25	0.005	0.008	0.024	0.042	6.9	6.9	0.992	0.991	0.962	0.957	0.449	0.425	16.6	15.4	0.527	0.573
0.3	0.003	0.007	0.017	0.035	6.2	6.4	0.997	0.993	0.983	0.965	0.384	0.388	14.9	14.4	0.616	0.611
0.35	0.003	0.006	0.014	0.030	6.2	6.1	0.997	0.994	0.986	0.969	0.374	0.365	14.7	13.7	0.626	0.635
0.4	0.002	0.005	0.009	0.023	5.6	5.8	0.998	0.995	0.991	0.977	0.304	0.326	12.6	12.8	0.696	0.674
0.45	0.002	0.003	0.009	0.016	5.5	5.6	0.998	0.997	0.991	0.984	0.290	0.299	12.3	12.2	0.710	0.701
0.5	0.001	0.003	0.007	0.014	5.4	5.5	0.999	0.997	0.993	0.986	0.261	0.268	11.7	11.4	0.739	0.731

Table 12: Equally mixed scenario (5 null, 5 alternative), family-wise type I error rate calibration across various predictive probability (PP) thresholds. $B = 0$ represents the Bayesian design without information sharing, $B = 0.1$ represents the design with information sharing through MEMs.

	Null Baskets					*Alternative Baskets*		
PP Threshold	Rejection Rate	Family-wise Rate	Expected Size	Stop Rate	Prob. All Null Stop	Rejection Rate	Expected Size	Stop Rate
0	0.034	0.161	25.0	0.000	0.000	0.799	25.0	0.000
0.05	0.032	0.150	16.5	0.734	0.222	0.781	24.1	0.074
0.1	0.027	0.131	12.3	0.871	0.516	0.728	22.2	0.184
0.15	0.024	0.115	9.8	0.908	0.640	0.664	20.4	0.276
0.2	0.022	0.104	8.8	0.927	0.708	0.625	19.4	0.328
0.25	0.019	0.089	7.9	0.959	0.830	0.573	18.1	0.400
0.3	0.014	0.068	7.2	0.971	0.872	0.515	16.7	0.467
0.35	0.012	0.057	6.6	0.977	0.898	0.448	15.0	0.540
0.4	0.009	0.042	6.1	0.984	0.924	0.394	13.7	0.597
0.45	0.007	0.034	5.8	0.987	0.938	0.368	13.1	0.626
0.5	0.006	0.032	5.7	0.990	0.949	0.340	12.6	0.657

Table 13: Equally mixed scenario (5 null, 5 alternative), family-wise type I error rate calibration across various predictive probability (PP) thresholds for Simon's 2016 design.

References

1. Kaizer AM, Koopmeiners JS, Hobbs BP. Bayesian hierarchical modeling based on multi-source exchangeability. *Biostatistics* 2017; **19**(2):169–184.

2. Kaizer AM, Hobbs BP, Koopmeiners JS. A multi-source adaptive platform design for testing sequential combinatorial therapeutic strategies. *Biometrics* 2018; **74**(3):1082–1094.

3. Hobbs BP, Landin R. Bayesian basket trial design with exchangeability monitoring. *Statistics in Medicine* 2018; **37**(25):3557–3572.

4. Kaizer AM, Koopmeiners JS, Kane MJ, Roychoudhury S, Hong DS, Hobbs BP. Basket designs: Statistical considerations for oncology trials. *JCO Precision Oncology* 2019; **3**:1–9.

5. Kaizer AM, Koopmeiners JS, Chen N, Hobbs BP. Statistical design considerations for trials that study multiple indications. *Statistical methods in medical research* 2021; **30**(3):785–798.

6. Simon R, Geyer S, Subramanian J, Roychowdhury S. The bayesian basket design for genomic variant-driven phase ii trials. *Seminars in Oncology*, vol. 43, Elsevier, 2016; 13–18.

7. Simon R. Optimal 2-stage designs for phase-II clinical trials. *Controlled Clinical Trials* 1989; **10**:1–10.
